# Comparison of cisplatin and mitomycin C/5-FU as radiosensitisers in the treatment of locally advanced vulvar cancer: results of a retrospective, observational, single-institutional cohort study

**DOI:** 10.1007/s00432-022-04006-0

**Published:** 2022-04-22

**Authors:** Valerie Catherine Linz, Carina Schwanbeck, Slavomir Krajnak, Katharina Anic, Jörg Jäkel, Roxana Schwab, Marcus Schmidt, Heinz Schmidberger, Annette Hasenburg, Marco Johannes Battista

**Affiliations:** 1 Department of Gynaecology and Obstetrics, University Medical Centre of the Johannes Gutenberg-University Mainz, Langenbeckstraße 1, 55131 Mainz, Germany; 2 Department of Radiooncology and Radiotherapy, University Medical Centre of the Johannes Gutenberg-University Mainz, Langenbeckstraße 1, 55131 Mainz, Germany; 3 Department of Pathology University Medical Center, University Medical Centre of the Johannes Gutenberg-University Mainz, Langenbeckstraße 1, 55131 Mainz, Germany

**Keywords:** Vulvar cancer, Radiosensitiser, Cisplatin, Mitomycin C, 5-Fluorouracil, Outcome

## Abstract

**Purpose:**

We retrospectively investigated the widely used radiosensitisers cisplatin and mitomycin C/5-fluorouracil (5-FU) in patients with locally advanced vulvar cancer for outcome and toxicity.

**Methods:**

We screened the archive for patients treated with chemoradiation for vulvar cancer diagnosed between 01/2010 and 08/2021 at our institution. The impact of both radiosensitisers on prognosis was compared using Kaplan–Meier method and Cox-regression analysis.

**Results:**

One hundred and forty-three patients with vulvar cancer were screened. Twenty-nine patients received chemoradiation (mitomycin C/5-FU *n* = 14; cisplatin *n* = 12; others *n* = 3) as a primary, neoadjuvant or adjuvant treatment. Median follow-up was 15.5 months. Patients in the cisplatin group were older (mean age 54.4 vs. 70.7; *p* = 0.004). However, the mitomycin C/5-FU group had more advanced tumour stages. The 2-year recurrence-free survival (RFS) was comparable (44.5% vs. 33.3%; *p* = 0.932). The 2-year overall survival (OS) showed a numerical but not statistically significant difference in favour of the mitomycin C/5-FU group (59.7% vs. 31.7%; *p* = 0.37). 64.3% (9 out of 14) patients, who received mitomycin C/5-FU achieved clinical complete response (cCR) compared to 41.7% (5 out of 12) who received cisplatin (*p* = 0.505). Radiodermatitis was the most common adverse event in both groups (81%) and more severe in the mitomycin C/5-FU cohort. Myelotoxicity was frequently observed in both groups. Eighteen patients received an additional radiation boost with 10.0 (9–16) Gy and showed a significantly prolonged RFS (*p* = 0.027) and OS (*p* = 0.003).

**Conclusion:**

Mitomycin C/5-FU may be considered in the treatment of young and healthy patients with locally advanced vulvar cancer.

## Introduction

Vulvar cancer is a rare disease, which accounts for 5% of all gynaecological malignancies after cancer of the uterus, ovary and cervix (Alkatout et al. 2015). 45,240 patients were newly diagnosed and 17,427 patients died of vulvar cancer worldwide in 2020 according to the Global Cancer Statistics 2020 (Sung et al. 2021). In the last years, vulvar cancer has shown an increasing incidence especially in women under the age of 60 (Kang et al. 2017). Therefore, we are urgently in need of a better knowledge of this rare malignancy and its treatment options. Especially locally extended malignant tumours of the vulvar which might already affect the inguinal and/or pelvic lymph nodes require a multidisciplinary management and remain a clinical challenge (Han et al. 2000; Lupi et al. 1996). These patients often benefit from a chemoradiation and/or brachytherapy as a primary or neoadjuvant treatment to avoid radical surgery such as exenterative procedures (Rao et al. 2017; Tagliaferri et al. 2021). Chemotherapeutic agents should improve outcome by acting as a radiosensitiser to increase locoregional control and by treating distant micrometastases (Mak et al. 2011). In general, chemoradiation protocols are often based on the experience in other squamous cell cancers, especially in cervical and anal cancer. Concurrent chemotherapy and radiation reduced local relapse rate and improved disease-specific and overall survival (OS) in primary treatment for locally advanced vulvar cancer (Han et al. 2000; Rao et al. 2017). Recently, radiation therapy has improved in terms of local control and toxicity due to image-guided and intensity-modulated radiation therapy (IMRT) (Beriwal et al. 2006; Tagliaferri et al. 2021).

The impact of different radiosensitisers on the outcome of locally advanced vulvar cancer patients has been investigated in multiple, mostly small retrospective observational studies. Commonly used radiosensitisers were platinum derivatives, mitomycin C, 5-fluorouracil (5-FU), rarely bleomycin, and often in combination with each other (ESGO-Guidelines 2016). A recently published phase II study reported promising results with capecitabine as radiosensitiser (van Triest et al. 2021). In general, high response rates, improved local recurrence and survival rates were described in locally advanced vulvar cancer patients after chemoradiation with mitomycin C/5-FU or 5-FU and/or cisplatin (Cunningham et al. 1997; Eifel et al. 1995; Landoni et al. 1996; Moore et al. 2012, 1998; Tans et al. 2011). Because of the low incidence of vulvar cancer, these studies included 2 to 71 patients and yielded a wide range of efficacy and toxicity, most of which were published between 1985 and 2013 (ESGO-Guidelines, 2016). In the most recent studies, which included patients treated after 2000, IMRT and image-guided treatment were used as the current standard of care (Beriwal et al. 2006; Tagliaferri et al. 2021).

The first Gynecologic Oncology Group (GOG) phase II study that evaluated preoperative chemoradiation with cisplatin and 5-FU in patients with advanced vulvar cancer was published in 1998. 46.5% of the patients had no visible vulvar cancer after chemoradiation (Moore et al. 1998). Both response rates and local control as well as toxicity of the 5-FU plus cisplatin regimen were promising. Based on the experience from cervical cancer trials, the following GOG Phase II study in 2012 evaluated a less toxic chemoradiation with weekly single-agent cisplatin for primary treatment of locally advanced vulvar cancer to achieve higher complete clinical and pathologic response rates and improved local control rates (Mak et al. 2011; Moore et al. 2012). Complete clinical response (cCR) was seen in 64% of the patients with high pathologic response rates and acceptable toxicity (Moore et al. 2012). On the basis of this background, the European Society of Gynaecological Oncology (ESGO) currently recommends chemoradiation with preferably cisplatin (Oonk et al. 2017). Tans et al. showed a complete response rate of 72% with mitomycin C/5-FU and an acceptable toxicity profile (Tans et al. 2011). In general, mitomycin C/5-FU may be associated with significant morbidity and mortality, especially due to its myelotoxicity (Bartelink et al. 1997; Flam et al. 1996; Mulayim et al. 2004). Nevertheless, it is worthwhile to further evaluate the combination of mitomycin C/5-FU in terms of its efficacy and toxicity in the treatment of vulvar cancer.

As a referral centre of vulvar cancer patients and due to limited data in the literature, we investigated both radiosensitisers cisplatin and mitomycin C/5-FU in locally advanced vulvar cancer in a retrospective, observational cohort study for outcome and toxicity.

## Methods

### Study population

We searched the archive for patients treated with chemoradiation for pathologically confirmed squamous cell cancer of the vulva at our institution between 01/2010 and 08/2021. Inclusion criteria were histological diagnosis of squamous cell cancer of the vulvar regardless of the stage of disease, Eastern Cooperative Oncology Group (ECOG)-status, or purpose of treatment (primary, neoadjuvant and adjuvant). cCR was defined by the absence of visible vulvar cancer after chemoradiation. Long-term follow-up was performed by evaluation of patient’s clinical records, inquiries to the patient’s physician, and by telephone calls through August 2021.

### Treatment and toxicity data

The standard dose of cisplatin was defined as 40 mg/m^2^ d1, q7d. Cisplatin 6 mg/m^2^ d1–d5, weeks 1, 2, 5, and 6, was regarded as a so called low-dose regimen. 1000 mg/m^2^ 5-FU d1–d4 and 10 mg/m^2^ mitomycin C d1, weeks 1 and 5, were applied intravenously with standard premedication. Radiation therapy was given concurrently on the first day of the first cycle of chemotherapy.

Radiation therapy was administered to the vulvar, lower pelvis, and bilateral inguinal lymph nodes using IMRT. Radiation boost was applied to the primary tumour bed and one or both involved groins if applicable. Radiation therapy was given in the outpatient setting except during concomitant chemotherapy cycles. All patients were hospitalised for the administration of concurrent chemotherapy and were monitored weekly during the treatment course for acute toxicity. Adverse events were classified according to the Common Terminology Criteria for Adverse Events (CTCAE) Version 3.0.

### Statistics

Statistical analysis was performed with SPSS statistical software programme, version 27.0.1 (SPSS Inc, Chicago, IL, U.S.A.). Patients’ characteristics were given in absolute and relative frequencies (categorical data) as mean (± standard deviation (SD)) or as median with their interquartile ranges. Continuous data were reported as means and SD or median and interquartile range. We compared chemoradiation protocols, response rates and toxicities. Normal distribution was examined with the Shapiro–Wilk-test, followed either by Mann–Whitney-*U* test or *t*-test to detect significant differences. The frequency of distribution of categorical variables was compared with the Fisher’s exact test.

The Cox-proportional hazard regression model was used to determine the prognostic influence of established risk factors such as mean age, ECOG-status, stage of disease (according to International Federation of Gynecology and Obstetrics (FIGO) stage), and histological grade of differentiation. Furthermore, radiation boost and radiation dose were included in the Cox regression analysis. First, univariate Cox regression analysis for every single variable was performed. Then, variables with a *p*-value < 0.05 were included in the multivariable Cox regression analysis with variable selection by backward elimination. Kaplan–Meier estimates were used to describe recurrence-free survival (RFS) and OS after 2 years. Time points in months were the date of diagnosis leading to the indication of a chemoradiation until death (or recurrence) or last follow-up. Patients who were still alive (or without recurrence) and/or had incomplete data were censored. RFS included loco-regional recurrences and/or distant metastasis and death as an event. In the Cox regression model, hazard ratio (HR) and 95% confidence interval (CI) were used. The Log-Rank-Test was used to compare the curves. All tests were two-sided and a *p*-value of < 0.05 was considered statistically significant. Since no correction was made for multiple testing, the results were considered exploratory.

## Results

**Fig. 1:**
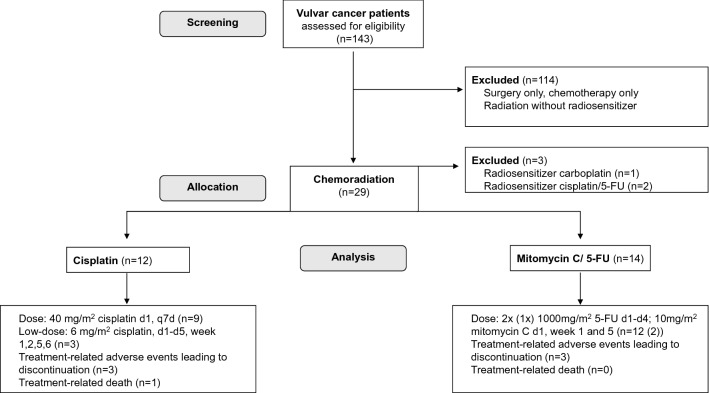
Consort diagram

A total of 143 patients with vulvar cancer were screened (Fig. 1). Twenty-nine patients received chemoradiation (cisplatin *n* = 12; mitomycin C/5-FU *n* = 14; others *n* = 3) as a primary or neo-/adjuvant treatment. Twelve patients of the cisplatin cohort, of whom 9 patients received the standard dose and 3 patients the low-dose regimen, and 14 patients of the mitomycin C/5-FU cohort were included in the final analysis.

Patients in the cisplatin group were significantly older than in the mitomycin C/5-FU group (mean age 70.7 vs. 54.4, *p* = 0.004) (Table 1). The mitomycin C/5-FU group showed higher tumour stages according to the FIGO classification (*p* = 0.023). Both groups showed no differences in terms of ECOG-status and histological grade. 64.3% of the patients who received mitomycin C/5-FU achieved cCR compared to 41.7% receiving cisplatin (*p* = 0.211) as radiosensitiser. Median radiation dose in total was 59.8 (54.15–66.4) Gy. 18 patients received an additional radiation boost with 10.0 (9–16) Gy. Radiation boost was applied 7 times in the cisplatin cohort and 11 times in the mitomycin C/5-FU cohort. Radiation boost was not performed in case of palliative treatment (*n* = 3), neoadjuvant treatment (*n* = 1), progressive disease (*n* = 1) and discontinuation of therapy due to adverse events (*n* = 3).

**Table 1:** Patients’ characteristics

Parameter	(*n* = 26) *n* (%) Chemoradiation	(*n* = 12) *n* (%) Cisplatin	(*n* = 14) *n* (%) Mitomycin/5-FU
Mean age [years] (± SD) (*p* = 0.004)	61.9 (**± **15.2)	70.7 (**± **12.27)	54.36 (**± **13.6)
Tumour stage (FIGO) before chemoradiation (*p* = 0.023)			
I	2 (7.7)	0 (0)	2 (14.3)
II	2 (7.7)	2 (16.7)	0 (0)
III	15 (57.7)	9 (75.0)	6 (42.9)
IV	7 (26.9)	1 (8.3)	6 (42.9)
ECOG before chemoradiation (*p* = 0.659)			
0	11 (42.3)	4 (33.3)	7 (50.0)
1	9 (34.6)	4 (33.3)	5 (35.7)
2	4 (15.4)	3 (25.0)	1 (7.1)
3	2 (7.7)	1 (8.3)	1 (7.1)
4	0 (0)	0 (0)	0 (0)
Histological grade			
G1	0 (0)	0 (0)	0 (0)
G2	19 (73.1)	9 (75.0)	10 (71.4)
G3	7 (26.9)	3 (25.0)	4 (28.6)
Histological type			
Squamous cell carcinoma	26 (100)	12 (100)	14 (100)
Type of treatment (*p* = 1.000)			
Primary chemoradiation	12 (46.2)	5 (41.7)	7 (50.0)
Neoadjuvant	2 (7.7)	1 (8.3)	1 (7.1)
Adjuvant	12 (46.2)	6 (50.0)	6 (42.9)
Median radiation dose [grey] (interquartile range) Mean radiation dose [grey] (± SD) (cumulative)	59.8 (54.15–66.4) 59.7 (**± **9.71)	59.4 (50.4–64.9) 60.21 (**± **11.6)	60.4 (55.7–66.4) 59.2 (**± **8.18)
Boost (p = 0.401)	18 (69.2)	7 (58.3)	11 (78.6)
Median boost dose [grey] (interquartile range) Mean radiation dose [grey] (± SD) (cumulative)	10.0 (9–16) 11.4 (**± **4.56)	9 (9–10) 9.571 (**± **3.26)	10.0 (9.4–16) 12.58 (**± **5.01)
Interstitial brachytherapy	3 (11.5)	2 (16.7)	1 (7.1)
Median follow-up * (interquartile range) mean [months] (± SD)	15.5 (9.25–54.5) 30.42 (**± **30.58)	15.5 (10.5–53.75) 30.25 (**± **31.52)	15 (3.75–54.5) 30.57 (**± **30.95)
Clinical response to treatment (*p* = 0.505)			
Complete	14 (53.8)	5 (41.7)	9 (64.3)
Partial	5 (19.2)	3 (25.0)	2 (14.3)
Stable	1 (3.8)	1 (8.3)	0 (0)
Progressive	3 (11.5)	2 (16.7)	1 (7.1)
Not assessable	3 (11.5)	1 (8.3)	2 (14.3)

*SD* standard deviation, *FIGO* International Federation of Gynecology and Obstetrics, *ECOG* Eastern Cooperative Oncology Group performance score

^*^Diagnosis before chemoradiation and last follow-up or death

**Fig. 2:**
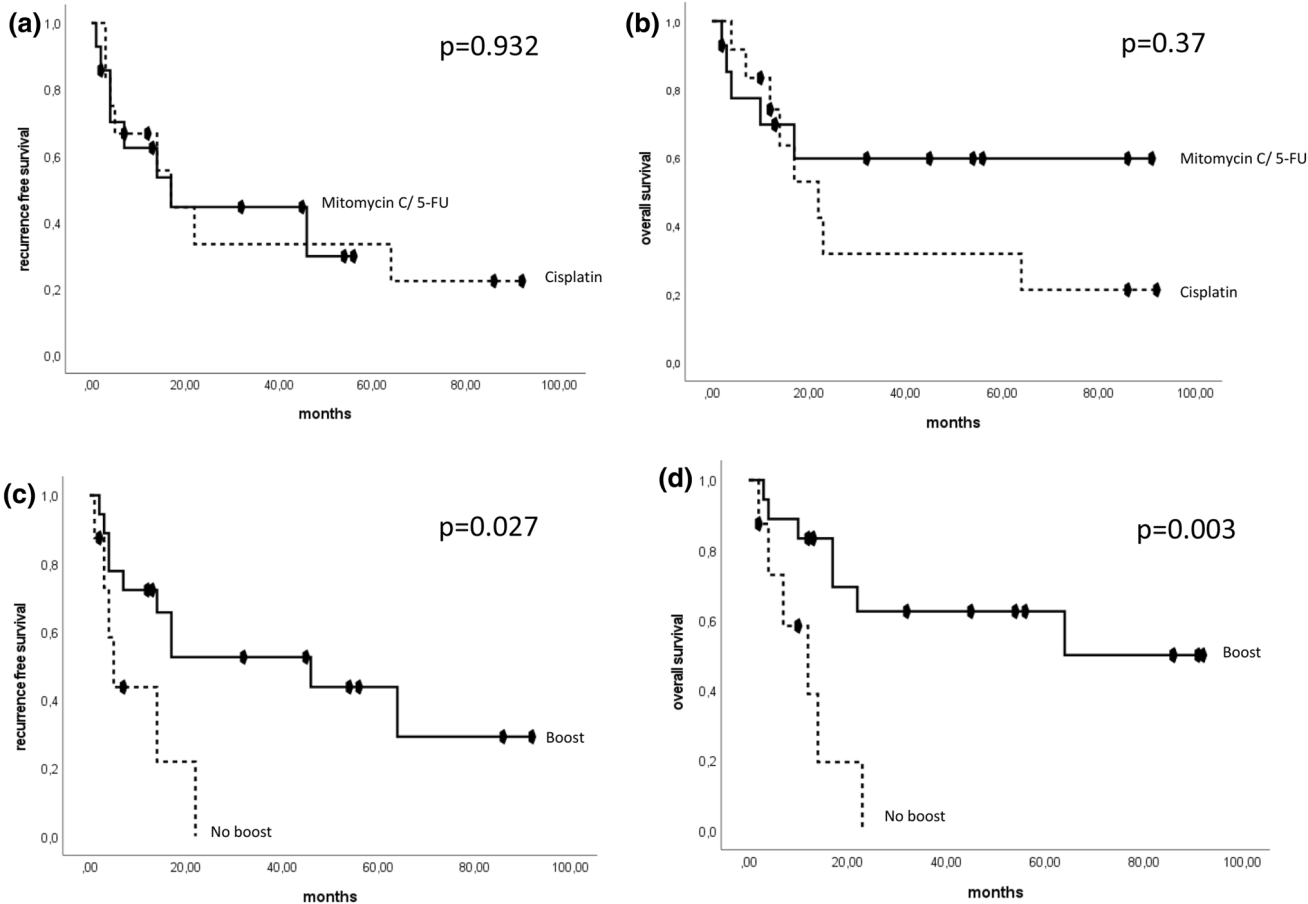
Kaplan–Meier curves showing RFS and OS for radiosensitiser cisplatin vs. mitomycin C/5-FU (**a**, **b**) and radiation boost (**c**, **d**). Time: months after diagnosis (chemoradiation)

The 2-year RFS was comparable between mitomycin C/5-FU and cisplatin (44.5% vs. 33.3%; *p* = 0.932). The 2-year OS showed a numerical but not statistically significant difference in favour of the mitomycin C/5-FU group (59.7% vs. 31.7%; *p* = 0.37) (Fig. 2a, b). Patients with a radiation boost showed a significantly improved RFS (*p* = 0.027) and an improved OS (*p* = 0.003) (Fig. 2c, d) compared to patients without boost. At 2 years, 52.5 (62.5%) of the patients who received a boost were still alive without recurrence compared to none of the patients who did not receive a radiation boost.

In the univariate Cox regression analyses, FIGO stage, histological grading, mean age, ECOG-status, and boost were associated with overall survival (Table 2). A better histological grading and boost were the only parameters associated with a longer RFS. In the multivariate analysis, histological grade and boost retained its significance for both RFS and OS (histological grade RFS: HR: 3.996; 95% CI [1.408–11.346]; histological grade OS: HR: 6.437; 95% CI [1.697–24.409]; boost RFS: HR: 0.295; 95% CI [0.095–0.916]; boost OS: HR: 0.208; 95% CI [0.062–0.703])**.** In the multivariate analysis, FIGO stage was also significantly associated with OS (FIGO OS: HR: 1.711; 95% CI [1.085–2.700]).

**Table 2:** Univariate and multivariate Cox-regression analyses for overall survival and recurrence-free survival

	Univariate—RFS			Univariate—OS			Multivariate—RFS			Multivariate—OS		
	HR	CI [95%]	*p* value	HR	CI [95%]	*p* value	HR	CI [95%]	*p* value	HR	CI [95%]	*p* value
Cisplatin	0.958	0.347–2.644	0.934	1.654	0.539–5.077	0.38						
Mitomycin C/5-FU	1.044	0.378–2.882	0.934	0.605	0.197–1.857	0.38						
Tumour stage—FIGO	1.178	0.924–1.502	0.185	1.526	1.019–2.283	**0.04**				1.711	1.085–2.700	**0.021**
Histological grade	3.771	1.357–10.480	**0.011**	3.707	1.230–11.176	**0.020**	3.996	1.408–11.346	**0.009**	6.437	1.697–24.409	**0.006**
Mean age	1.063	0.998–1.075	0.060	1.049	1.002–1.098	**0.039**				0.984	0.920–1.052	0.633
ECOG	1.633	0.966–2.761	0.067	2.267	1.241–4.140	**0.008**				1.565	0.721–3.398	0.258
Boost	0.322	0.108–0.953	**0.041**	0.199	0.061–0.648	**0.007**	0.295	0.095–0.916	**0.035**	0.208	0.062–0.703	**0.011**
Radiation dose in total	1.002	0.998–1.006	0.262	1.003	0.999–1.007	0.097						

Bold font: statistic significance was achieved (p < 0.05)

*HR* hazard ratio, *95% CI* confidence-interval, *FIGO* International Federation of Gynecology and Obstetrics, *ECOG* Eastern Cooperative Oncology Group performance score, *RFS* recurrence free survival, *OS* overall survival

Radiodermatitis was the most common adverse event in both groups (80.8% of all patients), and significantly more severe in the mitomycin C/5-FU group (Table 3). In addition, myelotoxicity was observed frequently in both groups. There was no statistically significant difference in the rates of leukopenia, thrombocytopenia and anaemia between both cohorts. However, two patients stopped the concurrent chemotherapy in the mitomycin C/5-FU group due to myelotoxicity of whom one had been kidney-transplanted twice. Treatment-related adverse events leading to discontinuation were similar (three cases in both groups). One patient, a 77-year old patient (ECOG-status: 3), in the cisplatin group died of treatment-related sequelae: a pneumonia after suffering from a superinfection of the radiodermatitis.

**Table 3:** Non-haematological and haematological toxicity

Parameter	Chemoradiation (*n* = 26) (%)	Cisplatin (*n* = 12) (%)	Mitomycin/5-FU (*n* = 14) (%)
Non-haematological toxicity			
Radiodermatitis, all (*p* = 0.006) CTCAE grade 3 or higher	21 (80.8) 4 (15.4)	10 (83.3) 0 (0)	11 (78.6) 4 (28.6)
Diarrhoea, all (*p* = 0.436) CTCAE grade 3 or higher	14 (53.8) 2 (7.7)	7 (58.3) 0 (0)	7 (50.0) 2 (14.3)
Infection (urinary tract, pneumonia, mucositis, and superinfection radiation burn)	9 (34.6)	3 (25.0)	6 (42.9)
Treatment-related adverse events leading to discontinuation	6 (23.1)	3 (25.0)	3 (21.4)
Treatment-related death	1 (3.8)	1 (8.3)	0 (0)
Haematological toxicity			
Leukopenia, all (*p* = 1.000) CTCAE grade 3 or higher	11 (42.3) 2 (7.6)	5 (41.7) 0 (0)	6 (42.9) 2 (14.3)
Thrombocytopenia, all (*p* = 1.000) CTCAE grade 3 or higher	5 (19.2) 2 (7.6)	2 (16.7) 0 (0)	3 (21.4) 2 (14.3)
Anaemia, all (*p* = 0.183) CTCAE grade 3 or higher	19 (73.1) 3 (11.5)	10 (83.3) 0 (0)	9 (64.3) 3 (21.4)
Anaemia, thrombocytopenia requiring transfusion	7 (26.9)	3 (25.0)	4 (28.6)
Treatment-related myelotoxicity leading to discontinuation	2 (7.6)	0 (0)	2 (14.3)

*P*-value is based on the Fisher’s exact test

## Discussion

We demonstrated that mitomycin C/5-FU as radiosensitiser showed a numerically but not statistically significant improved OS of locally advanced vulvar cancer compared with cisplatin. Of note, a clinically relevant high number of patients who had received mitomycin C/5-FU achieved cCR compared with cisplatin as radiosensitiser, although this was not statistically significant. In addition, radiation boost showed favourable outcome in terms of RFS and OS in locally advanced vulvar cancer patients. Regarding myelotoxicity, we could not proof a statistically significant difference in the rates of leukopenia, thrombocytopenia and anaemia between the two chemotherapy groups. Our cCR rates are comparable to the current literature. Tagliaferri and colleagues recorded in a recent systematic review a complete clinical response rate of 58.1% after chemoradiation regardless of the radiosensitiser for the primary treatment of locally advanced vulvar cancer or for patients who could not undergo surgery for medical reasons. Median 5-year disease-free survival and median 5-year OS were 45.6% and 49.9% which seemed to be higher than in our cohort (Tagliaferri et al. 2021). Furthermore, we expected higher rates of myelotoxicity and a significant morbidity and mortality in the mitomycin C/5-FU group regarding the literature (Bartelink et al. 1997; Flam et al. 1996; Mulayim et al. 2004). According to Mulayim and colleagues, 6 out of 17 patients, who were administered mitomycin C/5-FU as radiosensitisers in vulvar cancer had grade 4 neutropenia and 3 patients developed life-threatening neutropenic sepsis after the second cycle of chemotherapy (Mulayim et al. 2004). In contrast, we could not detect a statistically significant difference in the rates of leukopenia, thrombocytopenia and anaemia between concurrent chemotherapy with cisplatin or mitomycin C/5-FU in our cohorts. In 2017, a study reviewed squamous cell vulvar cancers in Waikato region, New Zealand, comparing toxicity grades of the radiosensitisers mitomycin C/5-FU in seven patients versus four patients, who received cisplatin (Dass and Kuper-Hommel 2017). None of the patients had a local recurrence. Severe toxicity was observed in the mitomycin C/5-FU group compared to the cisplatin group. However, the cisplatin cohort was much younger (median 50 years) compared to the mitomycin C/5-FU cohort (median 69 years), possibly resulting in an age selection bias. In contrast, in our study, the mitomycin C/5-FU cohort was significantly younger and might have tolerated the treatment better than the cisplatin cohort despite a higher FIGO stage. Higher age, female sex and a good ECOG-status were independent prognostic factors for non-haematological adverse events regarding the toxicity of 5-FU (Meta-Analysis Group In Cancer et al. 1998; Tebbutt et al. 2003). Even with a possible age selection bias in our study, our results are supported by the study of Tans and colleagues which also showed a good tolerability and an easy management of acute complications in 28 patients receiving mitomycin C/5-FU as a radiosensitiser, even in elderly patients (Tans et al. 2011). Diarrhoea, nausea, vomiting, and mucositis were the most common and dose-limiting toxicities associated with the use of 5-FU. However, 5-FU was associated with only 4% of grades 3–4 haematologic toxicity consisting mainly of neutropenia in case of continuous intravenous application (Meta-Analysis Group In Cancer; Lévy E, 1998). Concerning radiation therapy, radiation boosts were applied more frequently in the mitomycin C/5-FU group than in the cisplatin group. A boost was not performed because of reduced performance status, palliative intent, progressive disease or the maximum of radiation dose had already been applied before. This might has biased our positive effects of radiation boost on PFS and OS.

Our study has strengths and limitations. To our knowledge, this study is the first to compare survival data and adverse events between the radiosensitisers cisplatin and mitomycin C/5-FU in vulvar cancer with more than ten patients in each group. However, due to its retrospective nature, all conclusions of our single institutional cohort study should be interpreted with caution. In our study, there was no objective measurement of the clinical response and the groups were heterogenous, especially regarding the type of treatment and stage (FIGO I–IV). Moreover, treatment selection bias may be an important confounder in this study. Mitomycin C/5-FU was more likely administered to patients who were younger and showed fewer comorbidities or rapid tumour progression or had a longer life expectancy than the cisplatin cohort. Comorbidities were not taken into account, like for example the metabolic syndrome, which increases the risk for vulvar cancer with a hazard ratio of 1.78 (Nagel et al. 2011). In addition, a higher comorbidity score among vulvar cancer patients decreases the overall survival in these patients (Dass and Kuper-Hommel 2017; Ghebre et al. 2011). A further limitation is the short median follow-up period of 15.5 months.

Despite these limitations, our study provides valuable single-institution data on the largest vulvar cancer cohort so far that compares the use of mitomycin C/5-FU and cisplatin in chemoradiation. Younger patients may benefit from a chemoradiation with mitomycin C/5-FU in locally advanced vulvar cancer, but attention should be paid to its toxicity profile. Future multicentre and international efforts are needed to identify the most effective and least toxic agents and protocols for chemoradiation in the treatment of locally advanced vulvar cancer given its increasing incidence. A large collective of patients with vulvar cancer could allow further subgroup analyses in the future, e.g. regarding histopathological characteristics, to improve patient’s treatment. In the meantime, further evidence from individual institutions series will help to define and update guidelines for the management of patients with locally advanced vulvar cancer (Mulayim et al. 2004).

## Data Availability

All data generated or analysed during this study are included in this article and its tables and figures. Further enquiries can be directed to the corresponding author.
